# Comparative Study of Machine Learning for Predicting Compressive Strength in Oyster Shell Cementitious Composites

**DOI:** 10.3390/ma18235314

**Published:** 2025-11-25

**Authors:** Jinwoong Kim, Woosik Jang, Sunho Kang, Dongwook Kim, Heeyoung Lee

**Affiliations:** Department of Civil Engineering, Chosun University, 10 Chosundae 1-gil, Dong-Gu, Gwangju 61452, Republic of Korea; daw220@chosun.kr (J.K.); woosik@chosun.ac.kr (W.J.); lineho@chosun.ac.kr (S.K.); dwkim92@chosun.ac.kr (D.K.)

**Keywords:** oyster shells, machine learning, compressive strength prediction, optimal model

## Abstract

**Highlights:**

**What are the main findings?**

- Superplasticizer and cement had the strongest Pearson correlations with compressive strength.
- Random forest achieved the highest performance among the base models, with *R*^2^ = 0.8411.
- LightGBM was identified as the optimal model, effectively capturing nonlinear patterns in the data.

**What are the implications of the main findings?**

- Ensemble models accurately predict compressive strength of oyster shell cementitious composites.
- Insights into the relative importance and directional influence of input variables are provided.
- Reliable evaluation of the structural performance of composites incorporating waste oyster shells.

**Abstract:**

Annual oyster production in southern Korea reaches about 300,000 tons, generating an equivalent amount of waste oyster shells. Most are illegally dumped or stockpiled along coastlines, causing serious environmental issues. This study utilized machine learning to predict the compressive strength of oyster shell cementitious composites. A total of 336 datasets were used, including 189 experimental results and 147 from published literature. Input variables were water-to-cement ratio (W/C), silica fume, blast furnace slag, superplasticizer content, and curing conditions. Algorithm selection compared the performance of Ridge Regression, Support Vector Regression, Artificial Neural Network, and Random Forest (RF), with RF exhibiting the highest predictive performance (*R*^2^ = 0.8411). Ensemble algorithms including XGBoost, AdaBoost, Extra Trees, and LightGBM were optimized using GridSearchCV. Among these, LightGBM showed the best predictive capability with a mean absolute error of 3.1671, mean squared error of 17.8054, root mean square error of 4.2196, and *R*^2^ of 0.9042. SHAP analysis revealed that W/C and superplasticizer were the most influential variables. Oyster shells showed a negative correlation with sand, indicating the role of oyster shells as a substitute material. Thus, cementitious composites can maintain compressive strength and serve as sustainable construction materials when waste oyster shells are incorporated with appropriate admixtures.

## 1. Introduction

The southern coastal region of Korea produces approximately 300,000 tons of oysters annually, ranking second worldwide in output. This production generates approximately 300,000 tons of waste oyster shells every year and only about 30% are recycled. The remaining shells are dumped or stockpiled along coastlines, which leads to severe environmental problems [1,2,3]. In response, the Ministry of Oceans and Fisheries in Korea recently revised legislation to allow marine waste to be used as construction materials [4,5]. This regulatory change has stimulated research on recycling waste oyster shells as construction materials [6,7,8]. Waste oyster shells exhibit strength and permeability similar to those of natural sand, and the CaCO_3_ content of approximately 90% ensures that incorporation into cementitious composites does not interfere with hydration reactions. Owing to these characteristics, the potential of waste oyster shells as a sand replacement has been widely investigated [9,10,11].

The feasibility of waste oyster shells as a replacement for sand has been investigated previously [12,13,14]. Kim et al. [15] completely replaced sand with waste oyster shells in cementitious composites, achieving compressive strengths exceeding 30 MPa under both freshwater and sodium chloride solution curing conditions. Hong and Choudhury [16] incorporated up to 40% waste oyster shells as a partial sand replacement and reported that the high absorption rate and porous structure of waste oyster shells reduced concrete workability. Luo et al. [17] completely replaced natural aggregates with waste oyster shells and confirmed the applicability of the concrete for structural purposes. Lin et al. [18] found that 20% waste oyster shells enhanced resistance to dynamic impact. Further research has emphasized performance improvements through the combined use of waste oyster shells and supplementary materials. Cha et al. [19] revealed that admixtures containing SiO_2_ promoted the formation of C–S–H during hydration, enhancing compressive strength. The findings confirmed the feasibility of waste oyster shells as eco-friendly replacements for limestone and sand. Yang et al. [20] found that 10% waste oyster shells achieved comparable long-term strength to conventional concrete while improving resistance to carbonation and chemical attack. Yoon et al. [21] varied the waste oyster shell contents from 0% to 80% and observed a reduction in compressive strength with increasing replacement ratio owing to the porous and lightweight nature of the shells. Overall, these studies indicate that cementitious composites can maintain sufficient compressive strength and serve as sustainable construction materials when waste oyster shells are combined with appropriate admixtures.

Accurate measurement of the compressive strength of cementitious composites is essential to ensure structural safety and durability. However, traditional evaluation methods require long curing periods and repeated compressive strength testing, highlighting the need for reliable predictive techniques [22,23,24,25]. Machine learning has shown strong potential in predicting compressive strength by learning nonlinear relationships from diverse datasets [26,27,28,29]. Recently, many studies have applied machine learning to predict the compressive strength of cementitious composites [30,31,32,33,34]. In addition, recent research has adopted machine learning to predict structural member capacity and confinement behavior in reinforced concrete systems, which further indicates the applicability of data driven approaches in civil engineering [35,36,37,38]. Yeh [39] applied artificial neural networks (ANN) to predict the strength of high-performance concrete, finding stronger correlations than models based on regression. DeRousseau et al. [40] applied machine learning to field-placed concrete, revealing that tree-based models performed best when capturing nonlinear compressive strength behaviors. Jia et al. [41] used ensemble learning methods and confirmed through SHAP analysis that water-to-binder ratio and curing conditions were the most influential variables. Nguyen-Sy et al. [42] predicted uniaxial compressive strengths by incorporating mixture proportions and curing age as input variables, identifying tree-based ensemble models as superior. Yu et al. [43] proposed a self-learning support vector machine (SVM) model enhanced with cat swarm optimization, achieving higher predictive accuracy than conventional SVM models. Feng et al. [44] presented an adaptive boosting-based machine learning model to predict the compressive strength of concrete, which achieved higher predictive accuracy than ANN and SVM models. Inqiad et al. [45] compared Multi-Expression Programming, eXtreme Gradient Boosting (XGBoost), and Random Forest (RF) for predicting the compressive strength of self-compacting concrete at 28 days, with XGBoost achieving the highest correlation coefficient. Wang et al. [46] investigated RF, XGBoost, and Support Vector Regression (SVR) for mixtures containing fly ash and slag, concluding that XGBoost consistently outperformed other models in terms of the root mean square error (RMSE), *R*^2^, and mean absolute error (MAE). Wang et al. [47] further applied interpretable machine learning techniques, demonstrating high predictive performance (*R*^2^ = 0.928 for training and *R*^2^ = 0.966 for testing) with XGBoost, while SHAP analysis confirmed that the most influential variables were consistent with the established concrete theory. Abdelsattar et al. [48] implemented deep residual neural networks combined with variance-based sensitivity analysis for pozzolanic concrete, achieving an *R*^2^ of 0.94 on the test set and identifying silica fume and volcanic ash as critical contributors to strength development. These findings indicate that accurate prediction of compressive strength is essential to ensure the structural stability and durability of cementitious composites, and machine learning offers a reliable approach for this purpose.

**Figure 1:**
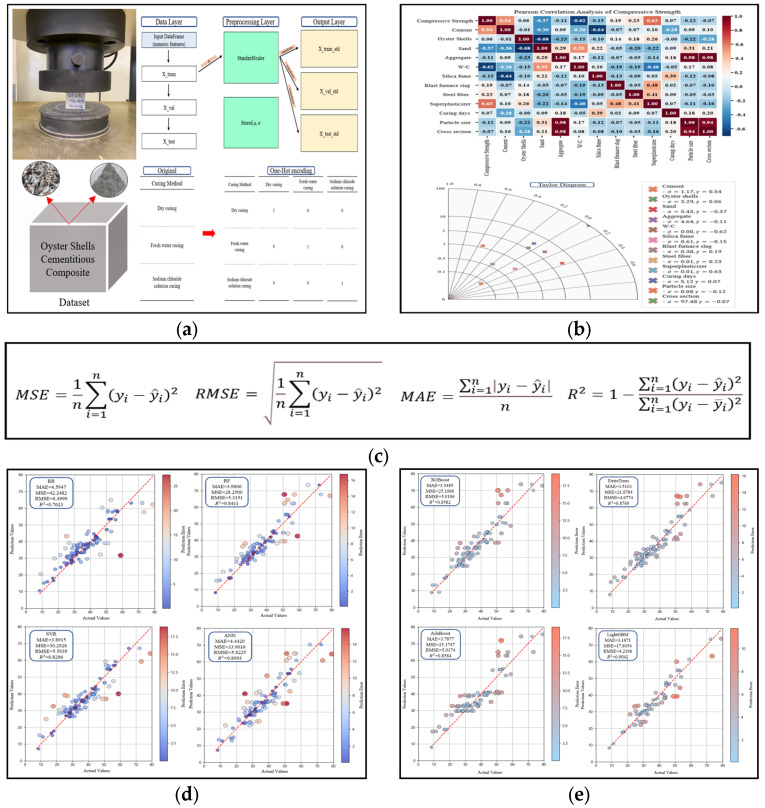
Research flowchart for predicting the compressive strength of oyster shell cementitious composites [15]: (**a**) Data preparation; (**b**) correlation analysis; (**c**) performance metrics; (**d**) Optimal model selection (RR, RF, SVR, ANN); (**e**) Emsemble model analysis (XGBoost, AdaBoost, ExtraTrees, LightGBM).

This study applied machine learning to predict the compressive strength of cementitious composites in which sand was replaced with waste oyster shells. The study was conducted in five stages, namely, data preparation, correlation analysis, performance evaluation, optimal model selection, and ensemble model analysis (Figure 1). In the data preparation process, datasets were constructed using input and output variables related to oyster shell cementitious composites. Standardization and one-hot encoding were applied during preprocessing [49,50,51]. This approach was utilized to ensure reliable prediction performance and identify key influencing variables. Through this process, the most effective detailed model for predicting the compressive strength of oyster shell cementitious composites was identified.

## 2. Experimental Program

### 2.1. Experimental Process

**Figure 2:**
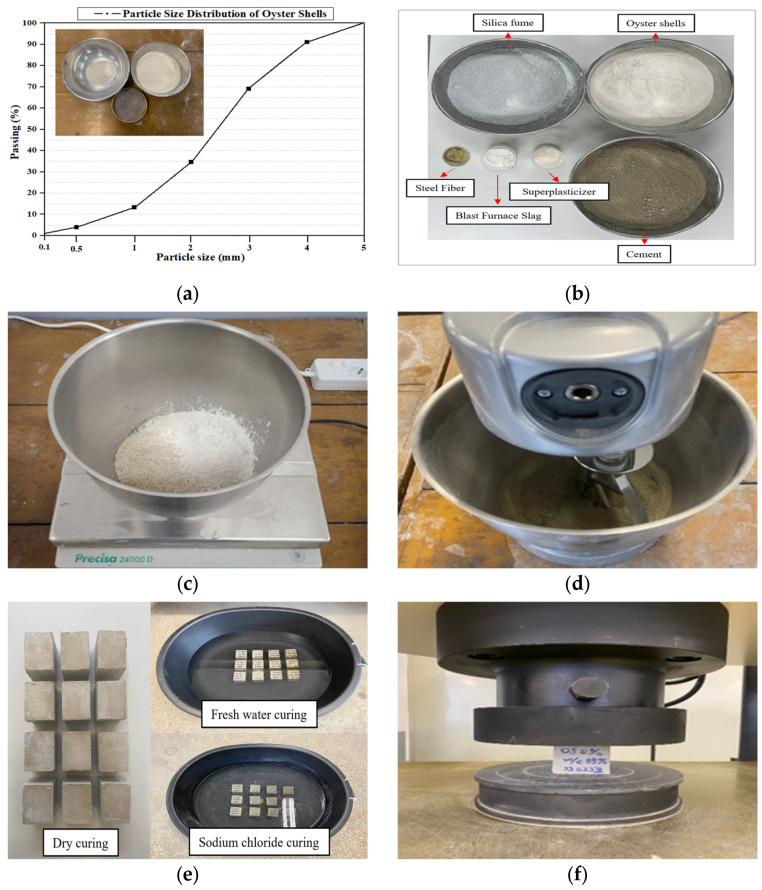
Experimental process [15]: (**a**) Sieve analysis of oyster shell; (**b**) material preparation; (**c**) measuring materials; (**d**) mixing; (**e**) curing; (**f**) measurement of compressive strength.

Figure 2 presents the experimental procedure for measuring the compressive strength of oyster shell cementitious composites. The waste oyster shells used in this study were obtained from Tongyeong, located on the southern coast of the Republic of Korea. To obtain the same particle size as Standard Sand No. 6 (0.35–0.7 mm), sieve analysis was performed using a #30 sieve with a mesh size of 600 μm (Figure 2a). Figure 2b shows the materials used in the oyster shell cementitious composite. Type 1 Ordinary Portland Cement was used, and chemical admixtures satisfying ASTM specifications were incorporated [52,53,54,55]. As shown in Figure 2c, cement, waste oyster shells, and admixtures were weighed prior to mixing. The measured materials were then placed into a mixing bowl and homogenized (Figure 2d). The fabricated oyster shell cementitious composite specimens had dimensions of 50 × 50 × 50 mm. Curing was conducted under three conditions—dry curing, freshwater curing, and sodium chloride curing (Figure 2e). Finally, compressive strength was measured using a Universal Testing Machine (UTM) with a capacity of 1000 kN, according to the ASTM C109 standards. The cross-sectional loading area was set to 2500 mm^2^, and the load was applied at a displacement rate of 1 mm/min. A low-capacity load cell range was selected on the UTM to ensure adequate sensitivity and accuracy for the small specimens. Figure 2f shows the compressive strength testing setup in accordance with ASTM C109 [56].

### 2.2. Datasets

**Figure 3:**
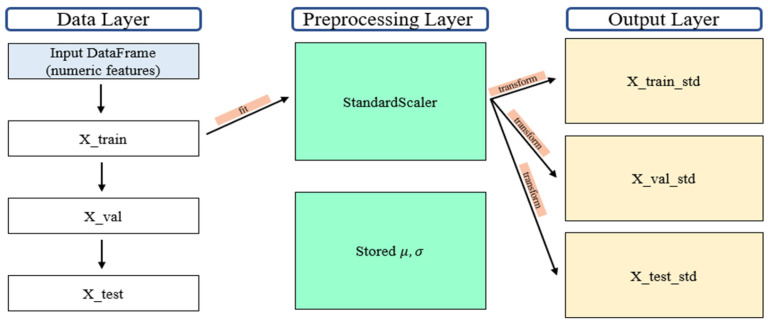
Standardization process of the dataset.

**Figure 4:**
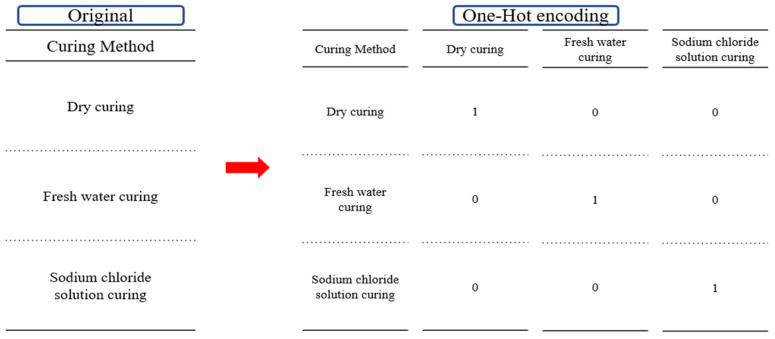
One-hot encoding process.

Compressive strength serves as a key indicator of structural stability and durability in cementitious composites, and therefore, this study designated compressive strength as the output variable. A total of 336 data points were used to construct the dataset, comprising 189 experimental results and 147 data points collected from published literature (Supplementary Material File). The input variables included basic mixture components such as cement, oyster shells, sand, aggregate, and water-to-cement ratio (W/C), as well as supplementary materials including silica fume, blast furnace slag, steel fiber, fly ash, and superplasticizer. Additional variables such as curing days, particle size, cross-sectional area, and curing conditions (dry curing, freshwater curing, sodium chloride curing) were incorporated to represent long-term strength development and environmental influences (Table 1). The compressive strength references for oyster shell cementitious composites are summarized in Table 2. All variables listed in Table 1 were used in the final model, and units for material proportions were expressed as mass ratio percentages. No missing data were present in the dataset. Therefore, no imputation was required. Cross-section was included because specimen size influences stress distribution and failure behavior during compression. Particle size was considered because packing density and pore structure associated with aggregate size affect hydration characteristics and compressive strength. To improve the stability of the learning process, standardization was applied to reduce scale differences among input variables (Figure 3). For categorical variables, one-hot encoding was applied to transform them into binary vectors (Figure 4). For example, the curing condition was divided into three categories (dry curing, freshwater curing, and sodium chloride curing), and each category was represented by a binary value of True (1) or False (0). This preprocessing enabled the model to effectively learn from both continuous and categorical variables. The dataset was divided into training validation and testing subsets, with 30% used as the test set and the remaining portion further split into training and validation sets to ensure generalization and prevent overfitting.

## 3. Research Method

### 3.1. Correlation Analysis

The Pearson correlation coefficient is a statistical measure used to evaluate the linear relationship between two continuous variables [60,61,62]. The coefficient ranges from −1 to +1, where values close to +1 indicate a strong positive linear correlation, values near 0 indicate no linear relationship, and values close to −1 indicate a strong negative linear correlation. The coefficient is defined as the covariance of the two variables divided by the product of their standard deviations, as shown in Equation (1). The Taylor diagram provides a graphical representation that simultaneously visualizes correlation coefficients and standard deviations between predicted and observed values. This method complements Pearson correlation analysis by enabling comparative evaluation of correlation strength and variance characteristics.

(1) $$r=\frac{\sum_{i=1}^{n}\left(x_{i}-\bar{x})(y_{i}-\bar{y}\right)}{\sqrt{\sum_{i=1}^{n}{\left(x_{i}-\bar{x}\right)}^{2}}\sqrt{\sum_{i=1}^{n}{\left(y_{i}-\bar{y}\right)}^{2}}}$$

Here, r denotes the Pearson correlation coefficient between variables x and y, n is the number of data points, $x_{i}$ and $y_{i}$ represent the individual data values, and $\bar{x}$ and $\bar{y}$ are the respective means of x and y.

**Figure 5:**
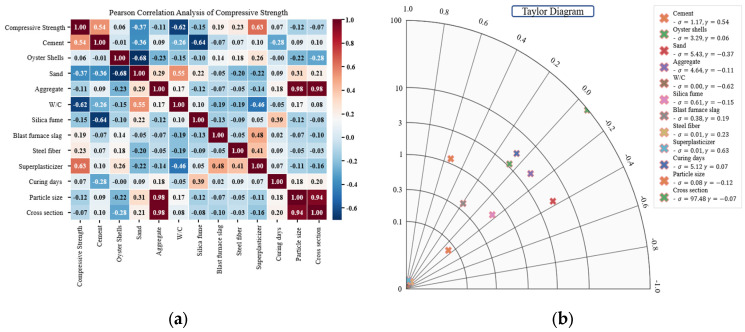
Correlation analysis results: (**a**) Pearson correlation heatmap; (**b**) Taylor diagram.

The correlation analysis results are presented in Figure 5. Superplasticizer (0.63) and cement (0.54) exhibited the strongest positive correlations with compressive strength (Figure 5a). This can be attributed to the role of superplasticizer in maintaining workability at low W/C ratios and enhancing hydration reactions, while cement contributes to strength development through the formation of calcium-silicate-hydrate (C–S–H). Steel fiber (0.23) showed a weak positive correlation. In contrast, W/C (−0.62) exhibited the strongest negative correlation, while sand (−0.37) and silica fume (−0.15) also showed negative correlations. A particularly strong negative correlation (−0.68) was observed between oyster shells and sand, confirming the role of oyster shells as substitute materials. The Taylor diagram further supported these findings, indicating that superplasticizer and cement were associated with high correlation coefficients and stable variance (Figure 5b). In contrast, W/C and sand exhibited negative correlations and high variability, identifying these variables as key factors contributing to strength reduction. Although oyster shells showed only a weak correlation with compressive strength, the strong negative correlation between oyster shells and sand was consistent with the Pearson analysis.

### 3.2. Background on Machine Learning Algorithms

**Figure 6:**
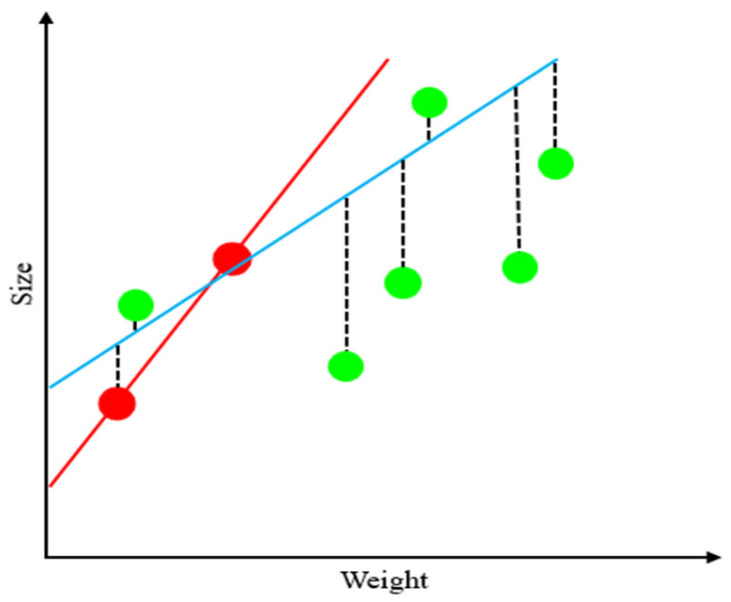
Ridge Regression (RR) architecture.

**Figure 7:**
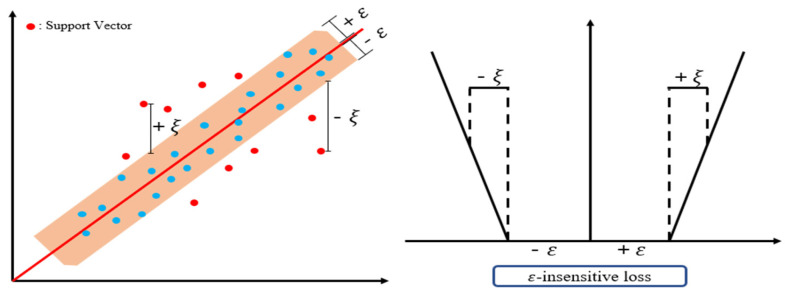
Support Vector Regression (SVR) architecture.

**Figure 8:**
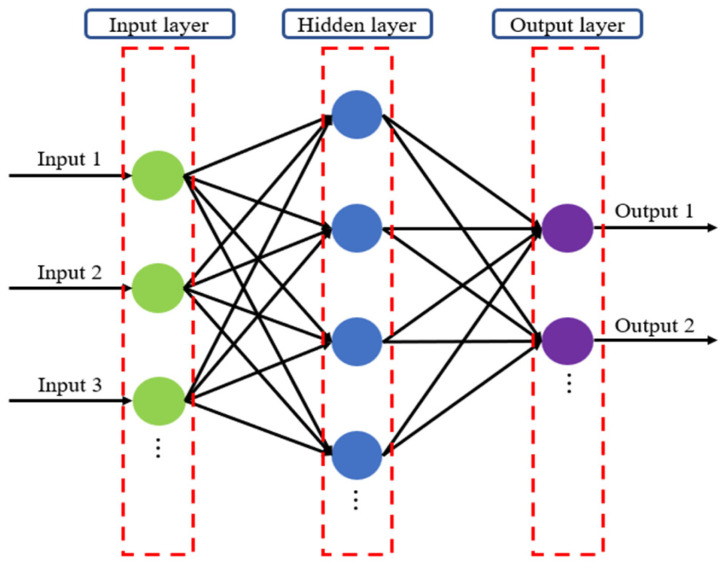
Artificial Neural Network (ANN) architecture.

**Figure 9:**
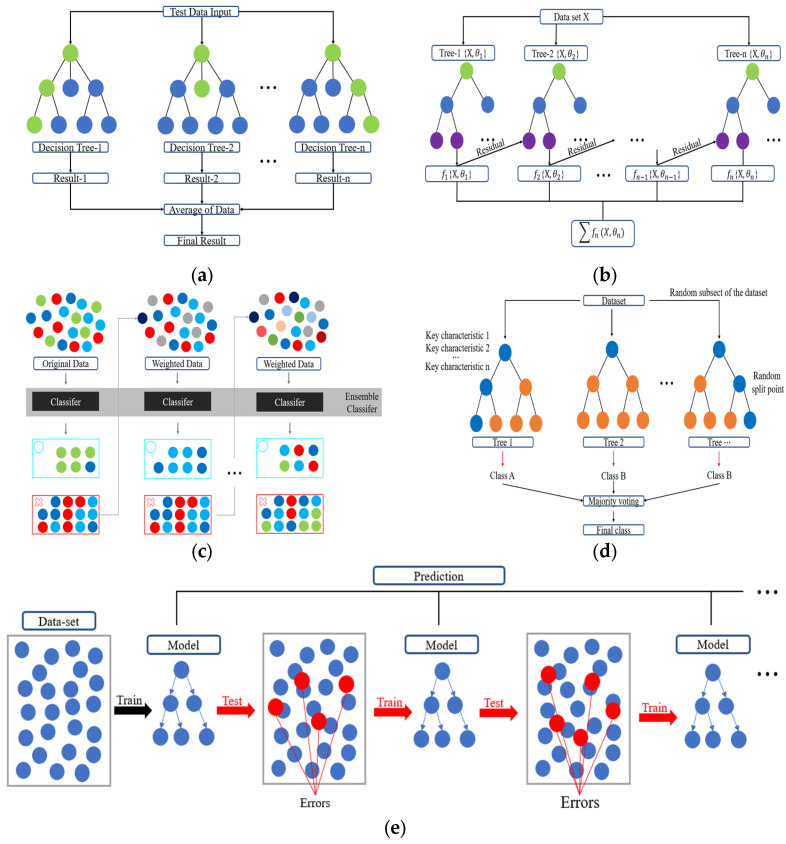
Architectures of ensemble learning models: (**a**) Random Forest (RF); (**b**) XGBoost; (**c**) Adaboost; (**d**) Extra Trees; (**e**) LightGBM.

This study utilized a range of regression and ensemble-based models to predict the compressive strength of oyster shell cementitious composites. The analysis was conducted in two stages. In the first stage, both regression models and ensemble models were compared to identify the optimal algorithm, and the characteristics of each model are summarized below. Ridge Regression (RR) applies L2 regularization to mitigate multicollinearity and ensure stable predictions [63]. By penalizing large regression coefficients, RR reduces the risk of overfitting when strong correlations exist among input variables, thereby improving performance (Figure 6). SVR uses kernel functions to effectively model nonlinear relationships. Input data are transformed into a high-dimensional feature space through kernel mapping, where SVR establishes an optimal decision boundary to reflect nonlinear dependencies [64]. Figure 7 presents the SVR architecture. ANNs are based on multilayer perceptrons and can model complex nonlinearities and interactions between input variables (Figure 8). In this study, the ANN model consisted of two hidden layers with 100 and 50 neurons using ReLU activation and Adam optimization, and early stopping was applied to prevent overfitting. Depending on the number of hidden layers and activation functions, ANN is capable of learning diverse patterns and achieving strong predictive performance when sufficient training data are available [65]. RF combines multiple decision trees to reduce variance and prevent overfitting [66]. In addition, RF provides the advantage of quantifying variable importance (Figure 9a).

In the second stage, additional ensemble models were evaluated to refine the selection of the optimal predictive model. XGBoost integrates boosting with regularization to deliver high predictive performance and computational efficiency [67]. The algorithm incorporates regularization terms and supports parallel learning, making the method effective for large-scale data processing (Figure 9b). Adaptive Boosting (AdaBoost, Figure 9c) improves performance by assigning higher weights to misclassified data and combining weak learners sequentially into a strong predictor [68]. Extra Trees incorporate randomness in both feature selection and data sampling during tree construction, which enhances computational efficiency and reduces variance (Figure 9d). Light Gradient Boosting Machine (LightGBM, Figure 9e) uses a leaf-wise tree growth strategy [69]. This approach enables fast training speed and high accuracy for large datasets, while also providing advantages in handling imbalanced data and optimizing memory usage [70]. The optimal model for predicting the compressive strength of oyster shell cementitious composites was determined by systematically comparing the performance of these regression and ensemble-based algorithms.

### 3.3. Performance Metrics

The predictive performance of oyster shell cementitious composites was evaluated using four statistical indices that include MAE, Mean Squared Error (MSE), RMSE, and the coefficient of determination (*R*^2^) [71,72]. For MAE, MSE, and RMSE, values closer to 0 indicate higher predictive accuracy, while for *R*^2^, values closer to 1 indicate stronger explanatory power. The calculation formulas are presented below.

MAE measures the average absolute difference between observed and predicted values, reflecting the mean prediction error (Equation (2)).

(2) $$MAE=\frac{1}{n}\sum_{i=1}^{n}\left|y_{i}-\hat{y_{i}}\right|,$$

where n is the number of data points, $y_{i}$ represents the observed value, and ${\hat{y}}_{i}$ is the predicted value.

MSE and RMSE evaluate the squared error between observed and predicted values. MSE represents the mean of squared errors (Equation (3)). RMSE is the square root of MSE, indicating the magnitude of average prediction error (Equation (4)).

(3) $$MSE=\frac{1}{n}\sum_{i=1}^{n}{\left(y_{i}-\hat{y_{i}}\right)}^{2}$$

(4) $$RMSE=\sqrt{\frac{1}{n}\sum_{i=1}^{n}{\left(y_{i}-\hat{y_{i}}\right)}^{2}}$$

*R*^2^ measures the proportion of variance in observed values explained by the model (Equation (5)).

(5) $$R^{2}=1-\frac{\sum_{i=1}^{n}{\left(y_{i}-\hat{y_{i}}\right)}^{2}}{\sum_{i=1}^{n}{\left(y_{i}-\bar{y}\right)}^{2}}$$

where n is the number of data points, $y_{i}$ represents the observed value, ${\hat{y}}_{i}$ is the predicted value, and $\bar{y}$ denotes the mean of the observed values.

## 4. Results and Discussion

### 4.1. Algorithm Performance Comparison and Optimal Model Selection

**Figure 10:**
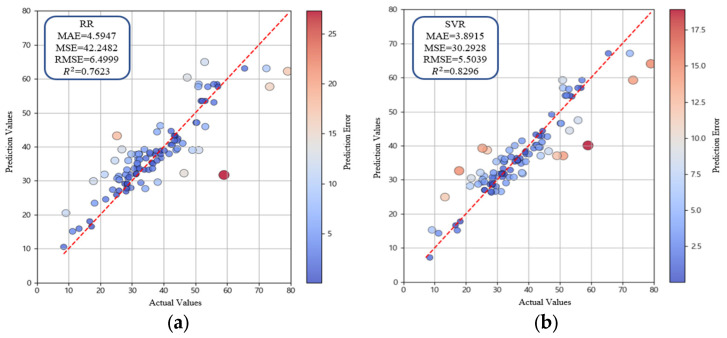

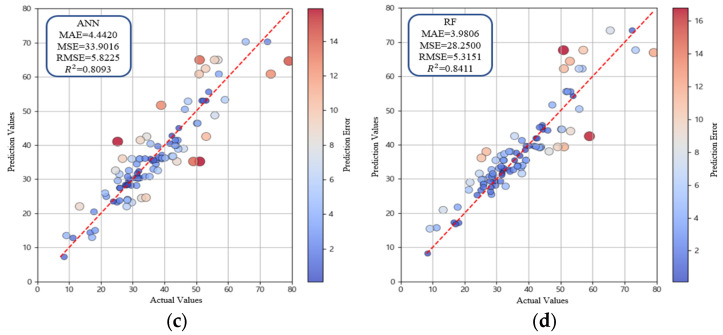
Performance comparison of machine learning algorithms for optimal model selection; (**a**) Ridge Regression (RR); (**b**) Support Vector Regression (SVR); (**c**) ANN; (**d**) Random Forest (RF).

The performances of RR, SVR, ANN, and RF are summarized in Table 3. Among these, RR exhibited the lowest predictive performance. This result can be attributed to linear assumptions of the model, which are insufficient to learn nonlinear relationships in the dataset (Figure 10a). By contrast, SVR effectively handled nonlinear data because of its kernel-based learning approach, achieving more stable performance compared with RR (Table 3, Figure 10b). ANN, despite the multilayer structure designed to capture complex nonlinear interactions, showed lower predictive accuracy than SVR owing to the limited dataset size and inherent structural constraints (Table 3, Figure 10c). In contrast, RF outperformed all other models (Table 3). The ensemble architecture of RF integrates multiple decision trees and effectively compensates for the limitations of individual regression models by modeling nonlinear interactions among variables and reducing variance, thereby achieving higher predictive accuracy than other regression-based models (Figure 10d). Cross validation score consistency confirmed that model performance differences were statistically reliable. Consequently, RF was identified as the most effective base algorithm and further analysis focused on additional ensemble models excluding RF to identify the optimal predictive approach for oyster shell cementitious composites.

### 4.2. Detailed Analysis of Ensemble Models

**Figure 11:**
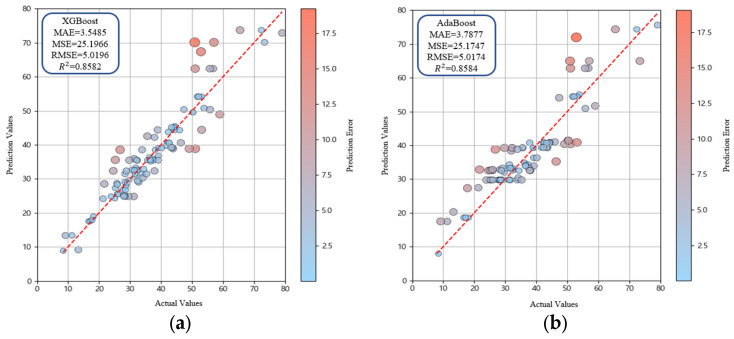

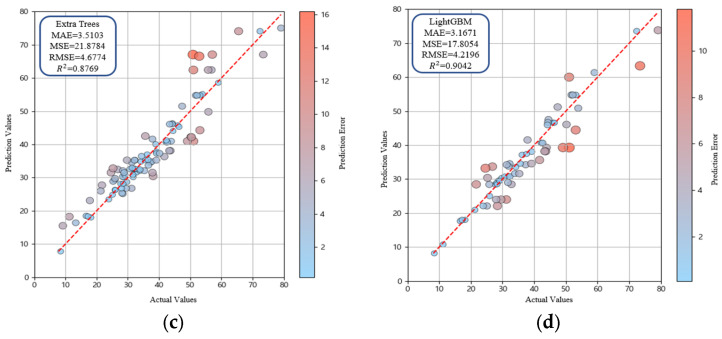
Performance comparison of ensemble learning algorithms: (**a**) XGBoost; (**b**) AdaBoost; (**c**) Extra Trees; (**d**) LightGBM.

**Figure 12:**
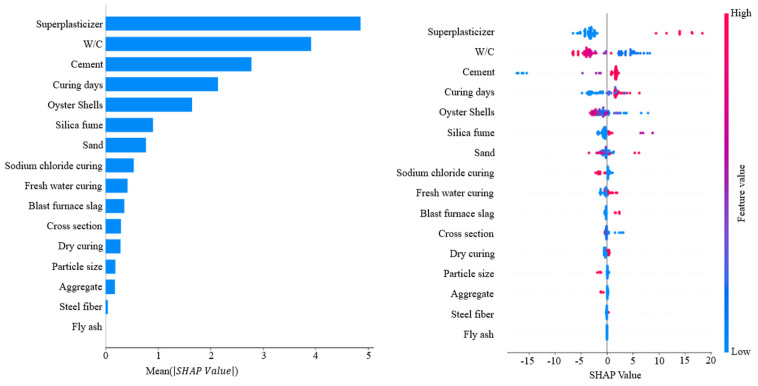
SHAP analysis results.

To optimize predictive accuracy, hyperparameter tuning was conducted using GridSearchCV [73,74]. Hyperparameter ranges were selected based on values commonly applied in previous studies and through an initial exploratory tuning phase to identify effective search boundaries. Parameters such as learning rate, maximum tree depth, and the number of estimators were adjusted, and cross-validation was applied to determine the optimal combinations. This process minimized overfitting and maximized model performance, and overfitting was additionally verified by analyzing training and validation loss trends and through assessment of stable performance on an independent test subset. The final hyperparameter settings are presented in Table 4, and the predictive results after tuning are summarized in Table 5. XGBoost improved prediction by iteratively correcting residuals of individual trees, but its performance was relatively lower than that of LightGBM. AdaBoost, which assigns higher weights to misclassified samples during iterative training, showed performance comparable to XGBoost. Extra Trees, which incorporates randomness into both feature selection and data sampling, reduced variance and delivered stable predictions. LightGBM showed superior performance to all other ensemble models (Figure 11). This enhanced performance was attributed not only to the leaf-wise growth strategy but also to efficient histogram-based feature grouping, regularization capability and high computational efficiency, which enhanced the model ability to represent nonlinear patterns in the dataset. Additionally, cross-validation score consistency confirmed that model performance differences were statistically reliable. Finally, SHAP analysis was conducted to quantify the contribution of each input variable and to interpret variable importance (Figure 12). Superplasticizer and W/C exhibited the highest SHAP values, indicating that these variables had a dominant influence on compressive strength prediction. Cement and curing days exhibited relatively high contributions, indicating key variables affecting compressive strength. Oyster shells showed an intermediate contribution and the strong negative correlation with sand confirmed the role of oyster shells as substitute materials. Distribution plots revealed that superplasticizer and cement generally had positive SHAP values, indicating a tendency to increase compressive strength, whereas W/C displayed negative SHAP values, confirming strength reduction at higher ratios. Curing days exhibited positive SHAP values, clearly showing strength improvement with longer curing periods. These results highlight that ensemble models not only predict the compressive strength of oyster shell cementitious composites with high accuracy but also provide interpretable insights into the relative importance and directional influence of input variables [75,76].

## 5. Conclusions

This study utilized a total of 336 datasets comprising 189 experimental results and 147 data points obtained from literature to predict the compressive strength of oyster shell cementitious composites. The datasets were preprocessed using standardization and one-hot encoding, and input and output variables were established accordingly. Correlations were analyzed through Pearson correlation coefficients and Taylor diagrams. The optimal algorithm was first determined by comparing RR, SVR, and ANN regression models with an ensemble model (RF). Subsequently, additional ensemble models including XGBoost, AdaBoost, Extra Trees, and LightGBM were analyzed to identify the most effective detailed model. Finally, SHAP analysis was performed to interpret the contribution, magnitude and direction of influence of each input variable.

- Pearson correlation analysis revealed that superplasticizer (0.63) and cement (0.54) exhibited the strongest positive correlations with compressive strength. In contrast, W/C (−0.62), sand (−0.37), and silica fume (−0.15) showed negative correlations. The strongest negative correlation (−0.68) was found between oyster shells and sand, confirming the substitutive relationship between these two materials. These trends were consistent with the Taylor diagram.
- In the comparison of predictive algorithms, RR showed the lowest performance with *R*^2^ = 0.7623. SVR (*R*^2^ = 0.8296) and ANN (*R*^2^ = 0.8093) partially captured nonlinear patterns, resulting in improved accuracy relative to RR. RF achieved the highest performance among the base models with *R*^2^ = 0.8411, showing the effectiveness of the model in reducing variance and mitigating overfitting. Accordingly, ensemble modeling was identified as the most suitable approach.
- Further ensemble analysis excluded RF and focused on XGBoost, AdaBoost, Extra Trees, and LightGBM. Hyperparameter tuning was performed using GridSearchCV. LightGBM achieved the highest predictive accuracy with MAE = 3.1671, MSE = 17.8056, RMSE = 4.2196, and *R*^2^ = 0.9042, confirming the superiority of the LightGBM model among the four models. Extra Trees produced stable performance by reducing variance through randomized splitting criteria, while XGBoost and AdaBoost improved accuracy using boosting techniques but showed lower performance compared with LightGBM. Overall, LightGBM was identified as the optimal detailed model, effectively capturing nonlinear patterns in the data.
- SHAP analysis confirmed that superplasticizer and W/C were the most influential variables while cement and curing days also contributed significantly. Oyster shells showed moderate influence and could function as a substitute material because negative correlation with sand was observed. Distribution plots indicated that superplasticizer and cement generally had positive SHAP values whereas W/C showed negative SHAP values. Curing days exhibited positive SHAP values, which confirms strength improvement with extended curing. These results indicate that oyster shell cementitious composites can maintain compressive strength and serve as sustainable construction materials when waste oyster shells are incorporated with appropriate admixtures.

## Figures and Tables

**Table 1:** Input variables with units and ranges for oyster shell cementitious composites.

Variables	Unit	Range of Data
Cement	%	25–100
Oyster Shell		0–227
Sand		0–300
Aggregate		0–267
Silica fume		0–25
Blast furnace slag		0–40
Steel fiber		0–1
Fly ash		0–40
Superplasticizer		0–0.5
W/C	-	0.3–0.5
Curing day	days	3–365
Particle size	μm	0.15–5
Cross-section	${mm}^{2}$	1600–7854
Curing Type	-	Dry curing
		Fresh water curing
		Sodium chloride curing

**Table 2:** Literature containing compressive strength data of oyster shell cementitious composites.

Index	Data Count	Reference
190–213	24	[19]
214–222	9	[57]
223–234	12	[58]
235–238	4	[14]
239–248	10	[11]
249–288	40	[6]
310–327	18	[59]
328–336	9	[8]

**Table 3:** Performance comparison of optimal machine learning algorithms.

Machine Learning Model	MAE	MSE	RMSE	$R^{2}$
RR	4.5947	42.2482	6.4999	0.7623
SVR	3.8915	30.2928	5.5039	0.8296
ANN	4.4420	33.9016	5.8225	0.8093
RF	3.9806	28.2500	5.3151	0.8411

**Table 4:** Hyperparameter settings of ensemble learning models.

XGBoost	colsample_bytree	0.7
	learning_rate	0.1
	max_depth	3
	n_estimators	200
	Subsample	0.7
Adaboost	max_depth	6
	learning_rate	0.1
	n_estimators	200
Extra Trees	Bootstrap	False
	max_depth	10
	max_features	0.8
	min_samples_leaf	1
	min_samples_split	2
	n_estimators	500
LightGBM	colsample_bytree	0.8
	learning_rate	0.05
	max_depth	−1
	min_child_samples	10
	num_leaves	31
	reg_lambda	1.0
	Subsample	0.8

**Table 5:** Performance comparison of ensemble learning models.

Machine Learning Model	MAE	MSE	RMSE	$R^{2}$
XGBoost	3.5485	25.1966	5.0196	0.8582
Adaboost	3.7877	25.1747	5.0174	0.8584
Extra Trees	3.5103	21.8784	4.6774	0.8769
LightGBM	3.1671	17.8054	4.2196	0.9042

## Data Availability

The original contributions presented in the study are included in the article or Supplementary Material, and further inquiries can be directed to the corresponding author.

## Supplementary Materials

The following supporting information can be downloaded at: https://www.mdpi.com/article/10.3390/ma18235314/s1.

## Abbreviations

The following abbreviations are used in this manuscript:

W/C	water-to-cement ratio
SF	Silica fume
BFS	Blast furnace slag
RR	Ridge recession
SVR	Support vector regression
ANN	Artificial neural network
RF	Random forest
MAE	Mean Absolute Error
MSE	Mean Squared Error
RMSE	Root Mean Squared Error
XGBoost	eXtreme Gradient Boosting
AdaBoost	Adaptive Boosting
LightGBM	Light Gradient Boosting Machine
